# Effect of *in vivo* neutralization of tumor necrosis alpha on the efficacy of antibiotic treatment in systemic *Salmonella enterica* infections

**DOI:** 10.1093/femspd/ftx002

**Published:** 2017-01-13

**Authors:** Omar Rossi, Andrew J. Grant, Pietro Mastroeni

**Affiliations:** Department of Veterinary Medicine, University of Cambridge, Cambridge CB3 0ES, UK

**Keywords:** TNF-alpha, *Salmonella*, antibiotic, *in vivo*, ampicillin, ciprofloxacin

## Abstract

Immunity can co-operate with antibiotics, but can also antagonize drug efficacy by segregating the bacteria to areas of the body that are less accessible to antimicrobials, and by selecting for subpopulations with low division rates that are often difficult to eradicate. We studied the effect of an anti-inflammatory/immunosuppressive anti-TNFα treatment, which accelerates bacterial growth in the tissues and inhibits or reverses the formation of granulomas, on the efficacy of ampicillin and ciprofloxacin during a systemic *Salmonella enterica* infection of the mouse. The anti-TNFα treatment neither precluded nor enhanced the efficacy of antibiotic treatment. However, the anti-TNFα treatment rendered the animals susceptible to the rapid relapse of the infection seen after cessation of the antibiotic treatment. Reactivation of an established infection, due to late administration of anti-TNFα antibodies, could be successfully controlled by antibiotics, but full clearance of the bacterial load from the tissues was not achieved. We conclude that the lack of TNFα does not preclude the efficacy of antibiotic treatment and must be monitored with care due to post-treatment relapses. Combinations of anti-cytokine compounds and antibiotic molecules may not be the best way to treat persistent infections with intracellular bacteria like *Salmonella*.

## Teaser

Effect of *in vivo* neutralization of tumor necrosis factor alpha on the efficacy of antibiotic treatment in systemic *Salmonella enterica* infections.

## INTRODUCTION

*Salmonella enterica* causes enteric systemic diseases (typhoid and paratyphoid fever), gastroenteritis and non-typhoidal septicaemia in humans and other animals worldwide and some serovars have zoonotic potential (Crump and Mintz 2010; Crump and Heyderman 2014; Crump *et al.*^2015^).

*Salmonella* infections can be difficult to treat. Persistence of the bacteria in the tissues and relapses can occur upon cessation of the treatment, especially in immunodeficient individuals (Crump, Luby and Mintz 2004; Gordon 2011; Okoro *et al.*^2012^; Klemm *et al.*^2016^). This is a grave medical problem especially in areas of the world where comorbidities such as malaria, HIV and malnutrition impair the immune system leading to higher incidence of both acute and recurrent bacterial infections, despite appropriate antimicrobial therapy (Gordon *et al.*^2002^). Better approaches to clear chronic infections are needed as these lead to disease reservoirs that are detrimental to human and veterinary medicine and can favor the selection of antimicrobial-resistant populations.

Despite the emergence of new multidrug-resistant bacterial isolates and the fact that we are losing many of our front-line antimicrobials, with very few new drugs currently in the pipeline (Cooke and Wain 2004; Spellberg *et al.*^2008^; Koirala *et al.*^2012^), resistance to the action of antibiotics and treatment failures cannot always be ascribed to the fact that the bacteria carry antimicrobial resistance genes. In fact, difficulties in treating infection and recurrent relapses occur despite the fact that the bacteria retain sensitivity to the antimicrobial used for the treatment of the patient (Gordon *et al.*^2002^; Okoro *et al.*^2012^). This generates situations where drugs that are highly effective *in vitro* are less effective *in vivo.* The reasons for these discrepancies are difficult to explain using traditional pharmacokinetics and pharmacodynamics parameters. Privileged sites that are poorly accessible to antibiotics, dormant non-replicative status of the bacteria and lack of cooperation between immunity and antimicrobials have all been inferred to be plausible causal factors in poor therapy outcome. This dictates the need for research into innovative strategies that can improve targeting of the bacteria within the tissues of animals and/or can modulate the growth rate of the pathogens to make them more vulnerable to treatment (Harish and Menezes 2011; Menezes *et al.*^2012^).

Successful antimicrobial treatment of systemic *Salmonella* infections often relies on the cooperation between drugs and immune effectors (Maskell and Hormaeche 1986; Gordon *et al.*^2002^; Mastroeni *et al.*^2003^; Crump, Luby and Mintz 2004; Gordon 2011; Okoro *et al.*^2012^). Host immunity can co-operate with antimicrobials by controlling growth and spread of the pathogens. However, it can also antagonize drug efficacy by segregating the bacteria to areas that are less accessible to antimicrobials, and by promoting the emergence of subpopulations with low division rates that are difficult to eradicate with drug treatment.

For example, the formation and persistence of inflammatory multicellular pathological lesions in the tissues (e.g*.* granulomata, abscesses), that is mediated by inflammatory cytokines (Mastroeni *et al.*^1991^; Mastroeni, Villarreal-Ramos and Hormaeche 1993; Mastroeni, Skepper and Hormaeche 1995; Everest, Roberts and Dougan 1998), can hamper tissue/intracellular penetration of antimicrobials and is likely to impact on the degree to which many intracellular pathogens (e.g*. Listeria*, *Mycobacteria*, *Brucella*, *Salmonella*) are exposed to antimicrobial drugs within the animal. The host inflammatory response and the escalation of the activation of phagocytes also create a hostile intracellular environment for many bacteria (Buchmeier and Heffron 1991; Vazquez-Torres *et al.*^2000^), resulting in bottlenecks that determine a marked heterogeneity in the division rates of individual microbial subpopulations. Reduced bacterial fission rates in turn decrease susceptibility to antimicrobials such as β-lactams and fluoroquinolones (Claudi *et al.*^2014^; Kaiser *et al.*^2014^).

Murine models have shown that tumor necrosis factor alpha (TNFα) is an important mediator in host resistance to *Salmonella* infections. TNFα mediates intracellular control of bacterial growth by phagocytes via enhancement of the localization of the NADPH oxidase to the phagosome and therefore appropriate delivery of reactive oxygen intermediates to the site of growth of intracellular bacterial growth (Vazquez-Torres *et al.*^2001^). TNFα is also necessary for the formation of multicellular pathological lesions, which restrain bacteria within discrete sites in the tissues and impede their uncontrolled systemic spread (Mastroeni, Skepper and Hormaeche 1995; Richter-Dahlfors, Buchan and Finlay 1997; Sheppard *et al.*^2003^). Genetic or immunological manipulations of mice which determine the lack of biologically active TNFα or TNFαR55 result in faster growth of *Salmonella* in the tissues and in their uncontrolled spread due to lack of lesions formation (Mastroeni *et al.*^1991^; Mastroeni, Villarreal-Ramos and Hormaeche 1992, 1993; Mastroeni, Skepper and Hormaeche 1995; Everest, Roberts and Dougan 1998). Neutralization of TNFα in the chronic phase of a *Salmonella* infection results in the regression of already established lesions and the reactivation of bacterial growth and spread in the tissues (Mastroeni, Villarreal-Ramos and Hormaeche 1993; Mastroeni, Skepper and Hormaeche 1995). Biologics based on anti-TNFα antibodies are widely used in humans for the treatment of autoimmune diseases and can lead to increased susceptibility to disease or reactivation of latent infections (Saraceno and Chimenti 2008; Mootoo *et al.*^2009^; Nanau and Neuman 2014; Shi and Liu 2014).

In this study, we used a murine model of systemic *Salmonella* infection to explore whether an anti-inflammatory/immunosuppressive treatment based on *in vivo* neutralization of TNFα would have a synergistic or detrimental effect on the course of treatment with ampicillin or ciprofloxacin. We therefore explored whether exacerbating bacterial growth and inhibiting their location/persistence within multicellular tissue lesions via *in vivo* administration of neutralizing anti-TNFα antibodies would result in a greater or lesser effect of the antibiotic treatment.

We investigated both the effects of TNFα neutralization early in the course of the disease and studied whether reactivation of an established infection would improve the reduction of the bacterial load in the tissue toward a more rapid and/or complete elimination of the infection.

## MATERIALS AND METHODS

### Antibodies and antimicrobials

Rabbit anti-murine TNFα serum was raised by Cambridge Research Biochemicals (Cambridge, UK) via immunization with recombinant murine TNFα (rmTNFα, Peprotech, London, UK). Five micrograms of rmTNFα was administered subcutaneously in Freund's complete adjuvant, followed by 5 and 25 μg booster doses in Freund's incomplete adjuvant after 28 and 56 days, respectively. IgG was purified from serum using Protein A Plus spin kit (Thermo Scientific, Waltham, MA, USA) according to the manufacturer's instructions, to a final concentration of 2 mg/mL. Samples were sterile filtered, and stored at –20°C until use. Purity of IgG was assessed by SDS-PAGE after Coomassie staining (bands of 50 and 23 kDa, corresponding to heavy and light chains, respectively), whereas specificity of anti-TNFα IgG was determined by western blot analysis. Rabbit IgG antibodies (Thermo Scientific) were used as control.

Ampicillin sodium salt and ciprofloxacin hydrochloride powders (Sigma Aldrich, Gillingham, UK) were resuspended in endotoxin-free water (Sigma Aldrich) to obtain the desired concentrations of antimicrobials and sterile filtered freshly before injections. The maximum upper dosage indicated for veterinary treatment of small rodent infections was chosen for our study (150 mg/kg in the case of ampicillin treatment and 20 mg/kg in the case of ciprofloxacin treatments).

### Infections and experimental schedules

Female innately resistant A/J *Scla11a^r/r^* mice (Hormaeche 1979) were purchased from Envigo laboratories UK and were used when over 7 weeks of age.

*Salmonella enterica* serovar Typhimurium JH3016 (Hautefort, Proenca and Hinton 2003), a chloramphenicol resistant derivative of SL1344 virulent strain with an intravenous (i.v.) LD_50_ of approximately 10 000 CFU for innately resistant mice, was used as the infection strain. For infections *Salmonella* were plated from glycerol stocks for 24 h at 37°C on Luria Bertani (LB) agar supplemented with chloramphenicol 20 μg/mL, before being inoculated (5–10 colonies) in LB media without antibiotic and cultured statically at 37°C for 16 h. The culture was then diluted in sterile phosphate-buffered saline to achieve the final cell density of ∼1 000 CFUs in 200 μL for i.v. injection. Inocula were enumerated by plating dilutions onto LB agar plates.

Antimicrobial treatments were administered by intraperitoneal (i.p.) injections (in 0.2 mL volume) at 12-h intervals for 5 days. Anti-TNFα IgG and control IgG antibodies were administered by i.v. injection, followed by further i.p. injections (0.4 mg in 0.2 mL volume) at 2–3 day intervals. In order to minimize the number of control mice, the experiments were conducted in parallel.

All animal experiments were performed in strict accordance with good animal practice as defined by the relevant international (Directive of the European Parliament and of the Council on the Protection of Animals Used for Scientific Purposes, Brussels 543/5) and local (Department of Veterinary Medicine, University of Cambridge) animal welfare guidelines and conducted under project license approved by the University of Cambridge Animal Welfare and Ethical Review Body, granted by the United Kingdom Home Office (license number PPL 80/2572), and performed in observance of licensed procedures under the United Kingdom Animals (Scientific Procedures) Act 1986.

### Enumeration of viable *Salmonella* in the organs and histology

Mice were killed by cervical dislocation, and spleens and livers were removed. Half of each spleen and liver was fixed in 10% formalin for histology and the other half used for bacterial counts. Organs were individually homogenized using Seward Stomacher 80 (Seward, Limited, Worthing, UK) in 5 mL of sterile water. Viable bacteria in the organs were enumerated by plating 10-fold dilutions onto LB agar plates, supplemented with chloramphenicol 20 μg/mL were appropriate. After fixation, livers and spleens were embedded in paraffin and 8-μm thick sections were stained with hematoxylin and eosin.

### Statistical analysis

Bacterial loads within organs at various time points/treatments were analyzed using ANOVA test and Tukey's correction for multiple comparisons. A probability value of ≤0.05 was considered statistically significant. The analysis was performed using GraphPad Prism 6 software.

## RESULTS

### Effect of antibiotic treatment on *Salmonella* infection in TNFα-depleted mice

**Figure 1.**
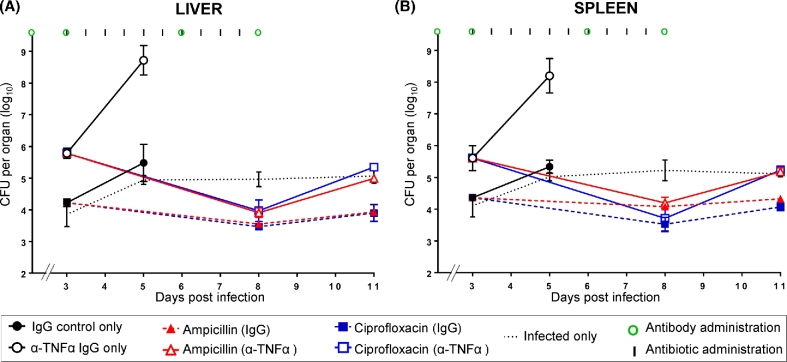
Effect of antibiotic treatment on a *Salmonella* infection in TNFα-depleted mice. Bacterial loads in livers **(A)** and spleens **(B)**. Mice were infected i.v. with ∼3.0 log_10_CFU of *Salmonella* Typhimurium at day 0. Different groups of mice received anti-TNFα IgG or control IgG antibodies at days 0, 3, 6 and 8 after infection (times of antibodies administration are illustrated by green circles). Ampicillin (red lines) and ciprofloxacin (blue lines) were administered to mice treated with anti-TNFα antibodies (open triangles and open squares respectively—solid lines) or control IgG (closed triangles and closed squares respectively—dashed lines) starting from day 3 post-infection for 5 days at 12-h intervals (times of antibiotics administration are indicated by black vertical lines). Control groups received either anti-TNFα antibodies or IgG only and no antibiotics (black open circles and black closed circles, respectively); a further group of mice (black dashed line) were only infected with *Salmonella* Typhimurium and did not receive either antibody or antimicrobial treatments. Results are expressed as the mean of log_10_ CFU values obtained in the organs ± standard deviations. Related statistical analyses are reported in Table S1.

We tested the efficacy of treatment with the antibiotics ampicillin and ciprofloxacin in mice that had been rendered immunodeficient prior to the commencement of the infection by *in vivo* neutralization of TNFα (Fig. 1 and Table S1, Supporting Information).

Different groups of four to five mice received anti-TNFα IgG or control IgG 2 h before being infected (day 0) with ∼3 log_10_ CFU of *Salmonella*. At 3, 6 and 8 days post-infection, the mice received additional injections of antibodies. TNFα neutralization induced the expected exacerbation of the infection in the spleens and livers (Mastroeni, Villarreal-Ramos and Hormaeche 1992; Mastroeni, Skepper and Hormaeche 1995). In the absence of antibiotic treatment, significantly higher viable bacterial counts were detected in the spleens and livers of anti-TNFα-treated mice on day 3 compared to mice receiving control IgG. By day 5, the infection progressed to high bacterial loads in the organs of anti-TNFα-treated mice, while mice treated with control IgG were able to better restrain bacterial growth.

Antibiotic treatment with ampicillin or ciprofloxacin was started on day 3 of the infection and continued for 5 days. Both antibiotics were able to abort the net growth rate of the bacteria in IgG-treated mice; however, only ciprofloxacin induced a statistical reduction in the bacterial load in the spleen and liver of control IgG-treated mice between day 3 and 8 of the infection. Cessation of antibiotic treatment on day 8 resulted in the presence/persistence of low bacterial loads in the tissues. Antibiotic treatment of TNFα-depleted animals prevented the escalation of the infection to high numbers that would otherwise lead to the rapid death of the animal. The treatment with either antibiotic induced a steeper decline of bacterial numbers in the organs of anti-TNFα-treated mice compared to IgG-treated mice, leading to similar bacterial loads in the two groups of animals by day 8 post-infection. Cessation of antibiotic treatment resulted in a relapse of bacterial growth in the organs of the TNFα-depleted mice.

Thus, immunodeficiency due to lack of TNFα does not preclude the possibility to treat a systemic *Salmonella* infection. In anti-TNFα-treated mice both ampicillin and ciprofloxacin prevented the uncontrolled escalation of the infection and induced a steep decline in viable bacterial numbers to levels similar to those observed after treatment of control animals. However, a relapse of the infection was seen in anti-TNFα-treated animals after cessation of treatment.

### Combined effect of anti-TNFα antibodies and antibiotic treatment on an established *Salmonella* infection

**Figure 2.**
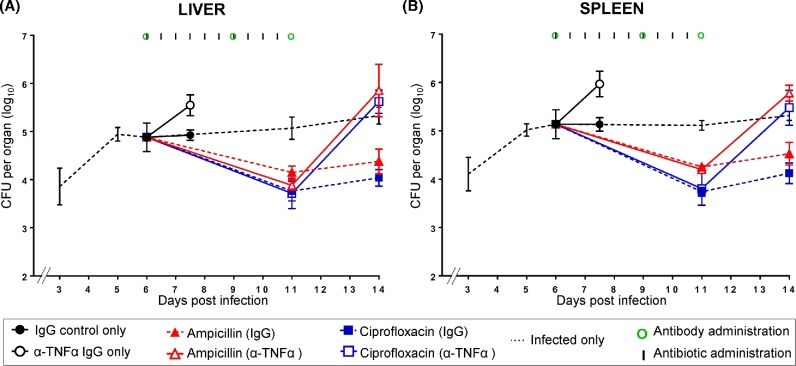
Combined effect of anti-TNFα antibodies and antibiotic treatment on an established *Salmonella* infection. Bacterial loads in livers **(A)** and spleens **(B)**. Mice were infected i.v. with ∼3.0 log_10_CFU of *Salmonella* Typhimurium and the infection was allowed to proceed until day 6. Different groups of mice received anti-TNFα IgG or control IgG antibodies at days 6, 9 and 11 after infection (times of antibodies administration are illustrated by green circles). Ampicillin (red lines) and ciprofloxacin (blue lines) were administered to mice treated with anti-TNFα antibodies (open triangles and open squares, respectively—solid lines) or control IgG (closed triangles and closed squares, respectively—dashed lines) starting from day 6 post-infection for 5 days at 12-h intervals (times of antibiotics administration are indicated by vertical lines). Control groups received either anti-TNFα antibodies or IgG only and no antibiotics (black open circles and black closed circles, respectively); a further group of mice (black dashed line) were only infected with *Salmonella* Typhimurium and did not receive either antibody or antimicrobial treatments. Results are expressed as the mean of log_10_CFU values obtained in the organs ± standard deviations. Related statistical analyses are reported in Table S2.

We tested whether the anti-inflammatory/immunosuppressive anti-TNFα treatment would have a synergistic or detrimental effect on an already established *Salmonella* infection when started simultaneously in a therapeutic combination with an antibiotic (Fig. 2 and Table S2, Supporting Information).

Mice were infected (day 0) with ∼3 log_10_ CFU of *Salmonella* and the infection was allowed to proceed until the suppression of bacterial growth was seen between days 5 and 6. At this point, different groups of mice received anti-TNFα IgG or control IgG, and two further injections of either anti-TNFα IgG or control IgG were given on days 9 and 11. The expected plateau in bacterial numbers was seen in mice receiving control IgG and no antibiotics (day 7.5). Whereas the expected exacerbation of the infection was seen in mice treated with anti-TNFα antibodies but no antibiotics.

A similar decline in bacterial numbers was seen in all organs of antibiotic-treated mice regardless of whether they received the anti-TNFα treatment or control IgG. However, upon cessation of antibiotic treatment a relapse of the infection was seen only in the anti-TNFα-treated mice.

Thus, simultaneous administration of antibiotics and anti-TNFα treatments during an established systemic *Salmonella* infection neither impairs nor enhances the rate at which antibiotics reduce bacterial numbers in the infected tissues. However, the anti-TNFα treatment renders the animals susceptible to relapse of the infection upon cessation of antibiotics.

### Antibiotic treatment after reactivation of a *Salmonella* infection using the anti-inflammatory/ immunosuppressive anti-TNFα treatment

**Figure 3.**
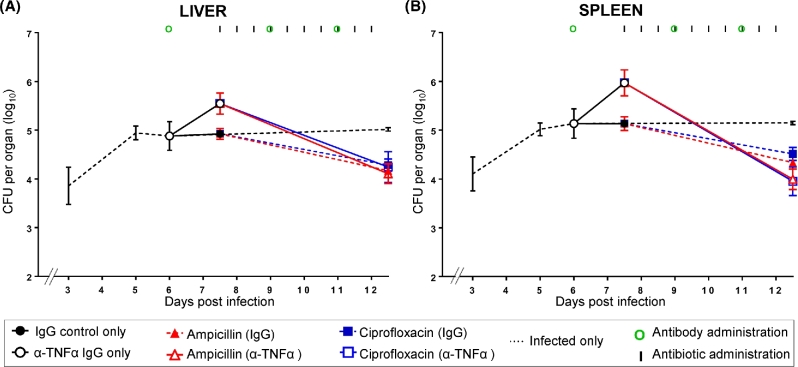
Antibiotic treatment after reactivation of a *Salmonella* infection induced by *in vivo* neutralization of TNFα. Bacterial loads in livers **(A)** and spleens **(B)**. Mice were infected i.v. with ∼3.0 log_10_CFU of *Salmonella* Typhimurium and the infection was allowed to proceed until day 6. Different groups of mice received anti-TNFα IgG or control IgG antibodies at days 6, 9 and 11 after infection (times of antibodies administration are illustrated by green circles). Ampicillin (red lines) and ciprofloxacin (blue lines) were administered to mice treated with anti-TNFα antibodies (open triangles and open squares, respectively—solid lines) or control IgG (closed triangles and closed squares, respectively—dashed lines) starting from day 7.5 post-infection for 5 days at 12-h intervals (times of antibiotics administration are indicated by vertical lines). Control groups received either anti-TNFα antibodies or IgG only and no antibiotics (black open circles and black closed circles, respectively); a further group of mice (black dashed line) were only infected with *Salmonella* Typhimurium and did not receive either antibody or antimicrobial treatments. Results are expressed as the mean of log_10_ CFU values obtained in the organs ± standard deviations. Related statistical analyses are reported in Table S3.

We tested whether the reactivation of bacterial growth and the regression of established multicellular pathological lesions determined by anti-TNFα antibodies (Mastroeni, Villarreal-Ramos and Hormaeche 1993; Mastroeni, Skepper and Hormaeche 1995) would increase the efficacy of treatment with ampicillin and ciprofloxacin (Fig. 3 and Table S3, Supporting Information).

Mice were infected as above and the infection was allowed to proceed until a suppression of bacterial growth was seen on day 6. One group of mice received the anti-TNFα treatment to induce the resurgence of bacterial growth that was confirmed by higher bacterial counts on day 7.5 compared to the mice that had received control IgG. Treatment with ampicillin or ciprofloxacin was started after the resurgence of bacterial growth, on day 7.5 of the infection. The treatment with either antibiotic resulted in a decline in bacterial numbers in the organs of both anti-TNFα and control IgG-treated mice. The decline in the spleens and livers of anti-TNFα-treated mice was steeper than what seen in the control group, but all mice had similar bacterial numbers in these organs on day 12.5 at the end of the antibiotic treatment.

Thus, reactivation of an infection due to anti-inflammatory/immunosuppressive anti-TNFα antibodies can be effectively treated with prompt administration of antibiotics.

However, therapeutic manipulation of a persistent infection via administration of anti-TNFα antibodies, which induce the reactivation of bacterial growth in the organs and the regression of established multicellular pathological lesions, does not lead to improved clearance of the infection load from the tissues. The residual bacterial numbers seen at the end of the antibiotic treatment are similar in the control group and in the anti-TNFα-treated group.

### Effect of antibiotic treatment on the histopathology of a *Salmonella* infection in TNFα-depleted mice

Treatment of animals with anti-TNFα antibodies leads to severe pathology due to impaired formation of multicellular pathological lesions at the foci of infection (Mastroeni, Skepper and Hormaeche 1995). We therefore tested whether, and to which extent, antibiotic treatment could ameliorate tissue pathology in infected animals receiving anti-TNFα antibodies.

**Figure 4.**
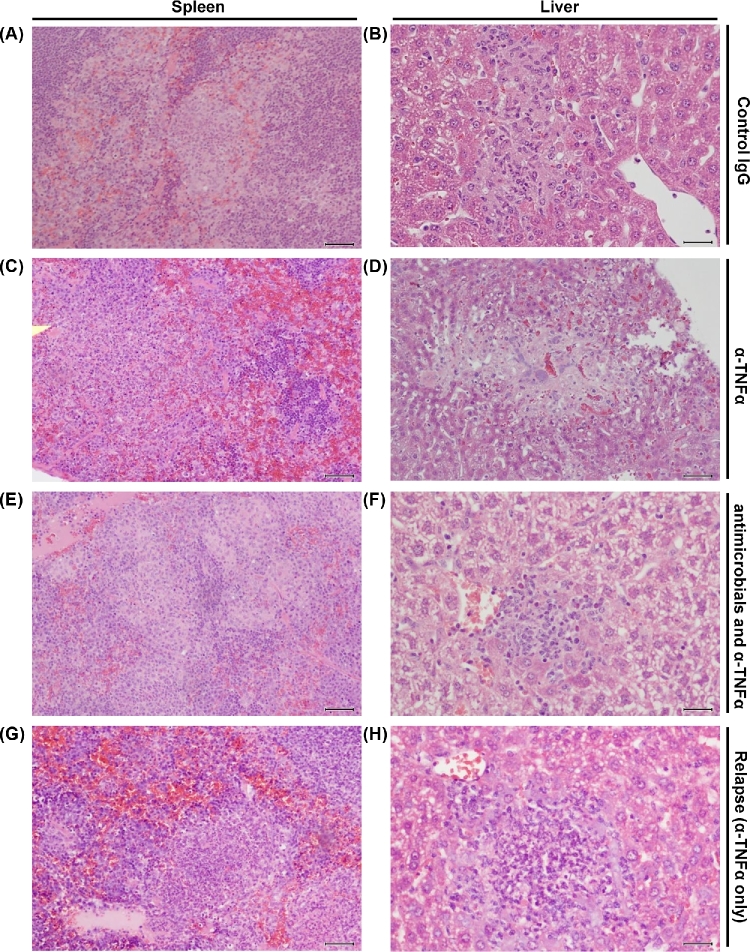
Effect of antibiotic treatment on the histopathology of TNFα-depleted mice infected with *Salmonella*. Representative images of hematoxylin-eosin stained tissue sections. Scale bar 30 μm. **(A** and **B)** Spleen and liver of mice treated with IgG antibody only (black closed circles in Figs 1–3) or untreated mice (dashed line in Figs 1–3) at day 5 post-infection. In these groups, the histology remained similar also at later time points. A similar histological picture was observed at all the time points studied in mice receiving control IgG and either antibiotic treatment (closed circles and closed triangles—dashed lines). (**C** and **D)** Spleen and liver of mice treated with anti-TNFα IgG (representative of what was observed on day 5 in Fig. 1 and on day 7.5 in Figs 2 and 3; black open circles). (**E** and **F)** Spleen and liver of anti-TNFα-treated mice at the end of the course of ampicillin administration (day 8 in Fig. 1, day 11 in Fig. 2, day 12.5 in Fig. 3; open triangles and red solid lines). A similar but less severe histological picture was seen in mice treated with anti-TNFα antibodies and ciprofloxacin (open squares and blue solid lines, see description in the main text). (**G** and **H)** Spleen and liver of anti-TNFα-treated mice after 3 days upon cessation of antimicrobial treatment (day 11 in Fig. 1, day 14 in Fig. 2). A similar, but less severe histological picture was observed in mice treated with anti-TNFα antibodies and ciprofloxacin (see description in the main text).

From day 5 of the infection, well-defined macrophage-rich pathological lesions and minimal or no pathological changes in the surrounding tissue areas were observed in spleens and livers of all the groups of mice treated with control immunoglobulins (Fig. 4A and B). Conversely, as expected (Mastroeni, Skepper and Hormaeche 1995), administration of anti-TNFα IgG resulted in severe pathology and tissue damage. The spleens of anti-TNFα-treated mice showed cellular depletion and large areas of hemorrhage in the red pulp, necrosis and polymorphonuclear leukocytes at various stages of degeneration (Fig. 4C). The livers showed focal lesions containing few mononuclear cells and abundant necrosis and cell debris (Fig. 4D).

Ampicillin therapy of anti-TNFα-treated mice resulted in a pathological picture that was different than the one induced by the anti-TNFα treatment only. In fact, cell depletion and hemorrhage of the red pulp were minimal, and well-defined large areas of macrophage infiltration with little necrosis were observed in the spleen (Fig. 4E). A qualitatively similar picture was seen in the spleens of mice treated with anti-TNFα antibodies and ciprofloxacin. In these mice, however, smaller macrophage-rich pathological lesions were seen and there was less disruption of the organization in the white pulp (data not shown). The livers of anti-TNFα-treated mice receiving either antibiotic showed small mononuclear lesions with little necrosis (Fig. 4F). Thus, ampicillin treatment reduces the severity of the histopathological picture normally seen in anti-TNFα-treated mice and ciprofloxacin almost completely inhibited/reversed the pathological changes cause by anti-TNFα antibodies. These results indicate that severe tissue damage and reduced/absent formation of multicellular pathological lesions normally seen in anti-TNF-treated mice infected with virulent bacteria (Mastroeni, Skepper and Hormaeche 1995; Everest, Roberts and Dougan 1998) can be prevented by antibiotic treatment. Therefore, multicellular lesions can form in the absence of TNFα provided that bacterial growth is kept under control and/or is suppressed by antibiotic administration.

Cessation of antibiotic administration in anti-TNFα-treated animals resulted in an increase in tissue pathology. In these mice, areas of necrosis were seen within the macrophage-rich pathological tissue lesions of the spleen (Fig. 4G) and liver (Fig. 4H) and cellular depletion and hemorrhage was present in the splenic red pulp. Thus, the immunosuppressive environment caused by *in vivo* neutralization of TNFα represents a persistent danger for the host once the infection is no longer controlled by antibiotics.

## DISCUSSION

In this study, we show that exacerbation or reactivation of *Salmonella* infections by anti-inflammatory/immunosuppressant treatment with anti-TNFα antibodies does not impair the ability of ampicillin or ciprofloxacin to reduce bacterial numbers in the tissues to levels similar to the ones obtained after treatment of immunocompetent animals. In anti-TNFα-treated animals, ampicillin or ciprofloxacin could prevent the escalation of the infection, which in the absence of antibiotic treatment would ultimately reach lethal bacterial numbers in the organs. The antibiotics could also reduce tissue pathology, which in anti-TNFα-treated mice would normally escalate toward widespread necrosis and within-organ hemorrhage.

The reduction in bacterial loads seen in anti-TNFα-treated mice was steeper than what was observed in control animals. This could be due to the higher division rates of the bacteria at the time the antibiotic treatment was implemented, as many antibiotics have higher efficacy on bacteria with higher fission rates. It is also possible that a larger proportion of the bacteria in the anti-TNFα-treated mice are in a location more accessible to the antibiotics. In fact *in vivo* TNFα inhibits the formation of multicellular pathological lesions that normally develop at the foci of infection and that restrain *Salmonella* within discrete areas within an organ (Mastroeni, Skepper and Hormaeche 1995; Richter-Dahlfors, Buchan and Finlay 1997; Sheppard *et al.*^2003^; Grant *et al.*^2012^). These lesions are poorly vascularized multicellular compartments, and would hinder the diffusion of antibiotics and their targeting to the bacteria (Mastroeni, Skepper and Hormaeche 1995; Everest, Roberts and Dougan 1998). The decline in bacterial numbers in mice treated with control IgG and antibiotics mice was small, likely due to low bacterial growth rates in the innately resistant mouse strain used in our experiments. Despite a steeper decline in bacterial numbers in mice receiving anti-TNFα IgG compared to mice receiving control IgG, the nadir in CFU counts that was reached at the end of the antibiotic treatment was similar in both groups of animals. Plausible explanations, to be tested in further studies, include the possibility that residual populations of bacteria either persist at sites that are not modified by the anti-TNFα treatment or retain very low division rates despite the immunosuppressive conditions in the TNFα-depleted animals. Histology showed the presence of well-defined areas of macrophage infiltration at the end of the course of antibiotics given to anti-TNF-treated mice. It is therefore possible that at the beginning of the antibiotic treatment, the lack of multicellular macrophage-rich lesions makes the bacteria more vulnerable to antibiotics, and hence the steep decline in CFU counts. As the bacterial numbers are progressively reduced by the antibiotic treatment in the TNFα-depleted animals, focal mononuclear lesions become more abundant and bacteria are possibly protected from the antibiotics within these lesions.

The bacteria that persist after antibiotic treatment are clearly not impaired in their ability to resume fast growth rates and they can therefore fully exploit the immunodeficient environment within the organs of anti-TNFα-treated animals upon cessation of antibiotic administration. These results provide an important warning for the management of antimicrobial treatments in patients undergoing anti-inflammatory or immunosuppressive therapies (steroids, anti-cancer agents, anti-cytokine biologics) and strongly indicate that the antimicrobial treatment should be prolonged until the effects of the immunosuppressant have fully regressed.

In this study, we have also tested whether reactivation/relapse of bacterial growth after full immunological control of the infection process can modify the outcome of the antibiotic treatment. We showed that although an anti-cytokine treatment can have potential dangers in a persistent infection due to induced resurgence of bacterial growth, the reactivation of the infection can be controlled successfully by prompt antibiotic treatment. However, the overall effect of treating an experimentally reactivated infection is similar to that seen in the treatment of a ‘non-reactivated’ persistent disease. This seems to indicate that combinations between anti-cytokine biologics and antibiotic molecules may have limited efficacy in the eradication chronic, persistent infections with intracellular bacteria like *Salmonella*.

## Supplementary Material

Supplemental material Supplementary data are available at *FEMSPD* online.
