# Supplementary material for: Effect of in vivo neutralization of tumor necrosis alpha on the efficacy of antibiotic treatment in systemic Salmonella enterica infections
Source: Pathog Dis. 2017 Jan 13;75(1):ftx002. doi: 10.1093/femspd/ftx002 (PMC5353993; doi:10.1093/femspd/ftx002)
Supplement: Supplemental material — Supplementary data are available at FEMSPD online. [file ftx002_supp.pptx]

## Slide 1
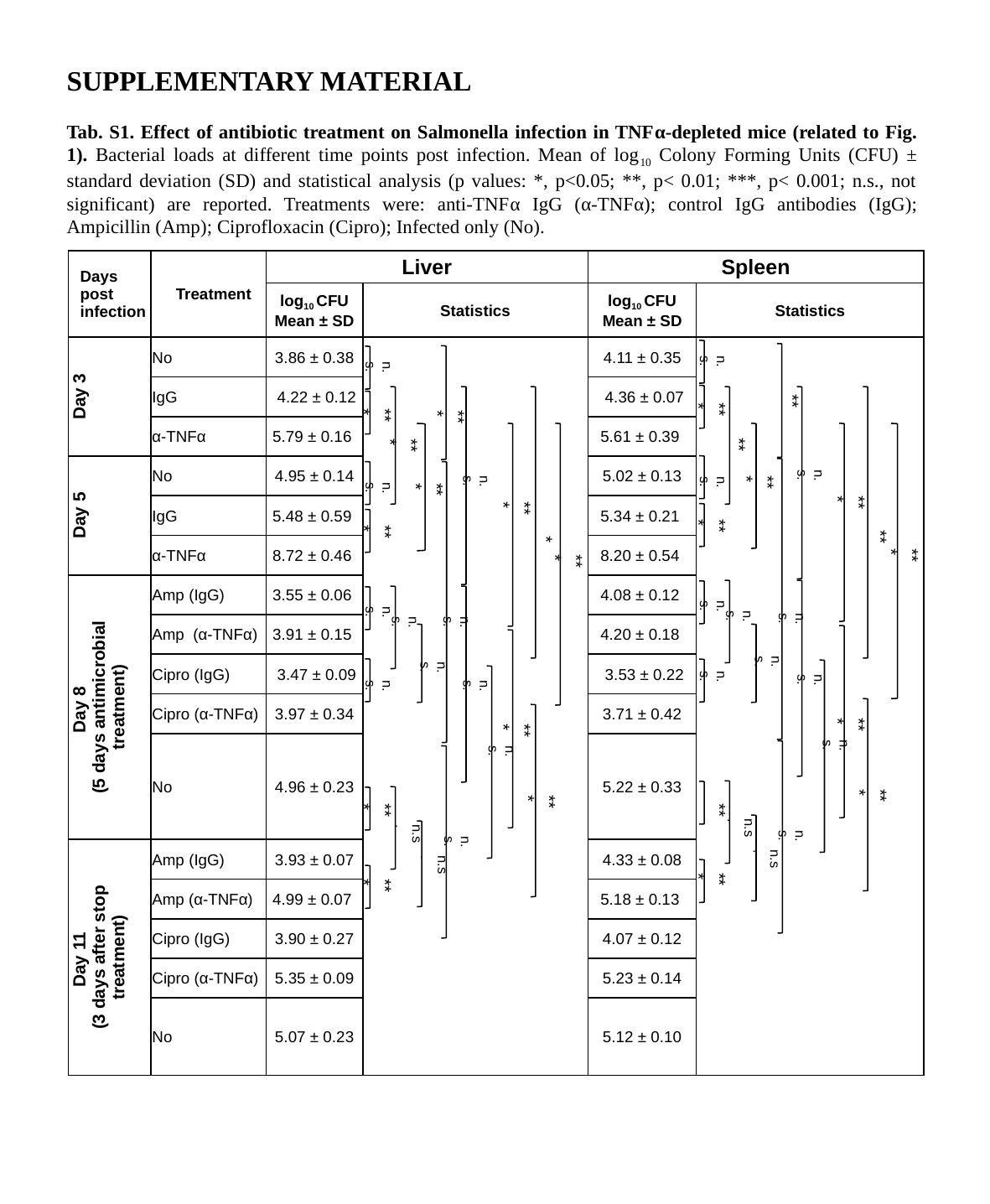

SUPPLEMENTARY MATERIAL
Tab. S1. Effect of antibiotic treatment on Salmonella infection in TNFα-depleted mice (related to Fig. 1). Bacterial loads at different time points post infection. Mean of log10 Colony Forming Units (CFU) ± standard deviation (SD) and statistical analysis (p values: *, p<0.05; **, p< 0.01; ***, p< 0.001; n.s., not significant) are reported. Treatments were: anti-TNFα IgG (α-TNFα); control IgG antibodies (IgG); Ampicillin (Amp); Ciprofloxacin (Cipro); Infected only (No).
| Days post infection | Treatment | Liver | | Spleen | |
| --- | --- | --- | --- | --- | --- |
| | | log10 CFU Mean ± SD | Statistics | log10 CFU Mean ± SD | Statistics |
| Day 3 | No | 3.86 ± 0.38 | | 4.11 ± 0.35 | |
| | IgG | 4.22 ± 0.12 | | 4.36 ± 0.07 | |
| | α-TNFα | 5.79 ± 0.16 | | 5.61 ± 0.39 | |
| Day 5 | No | 4.95 ± 0.14 | | 5.02 ± 0.13 | |
| | IgG | 5.48 ± 0.59 | | 5.34 ± 0.21 | |
| | α-TNFα | 8.72 ± 0.46 | | 8.20 ± 0.54 | |
| Day 8 (5 days antimicrobial treatment) | Amp (IgG) | 3.55 ± 0.06 | | 4.08 ± 0.12 | |
| | Amp (α-TNFα) | 3.91 ± 0.15 | | 4.20 ± 0.18 | |
| | Cipro (IgG) | 3.47 ± 0.09 | | 3.53 ± 0.22 | |
| | Cipro (α-TNFα) | 3.97 ± 0.34 | | 3.71 ± 0.42 | |
| | No | 4.96 ± 0.23 | | 5.22 ± 0.33 | |
| Day 11 (3 days after stop treatment) | Amp (IgG) | 3.93 ± 0.07 | | 4.33 ± 0.08 | |
| | Amp (α-TNFα) | 4.99 ± 0.07 | | 5.18 ± 0.13 | |
| | Cipro (IgG) | 3.90 ± 0.27 | | 4.07 ± 0.12 | |
| | Cipro (α-TNFα) | 5.35 ± 0.09 | | 5.23 ± 0.14 | |
| | No | 5.07 ± 0.23 | | 5.12 ± 0.10 | |
n.s.
n.s.
**
***
***
***
**
***
n.s.
n.s.
***
n.s.
n.s.
***
***
***
***
***
**
*
***
***
n.s.
n.s.
n.s.
n.s.
n.s.
n.s.
n.s.
n.s.
n.s.
n.s.
n.s.
n.s.
***
***
n.s.
n.s.
***
***
***
**
n.s.
n.s.
n.s.
n.s.
n.s.
n.s.
***
***

## Slide 2
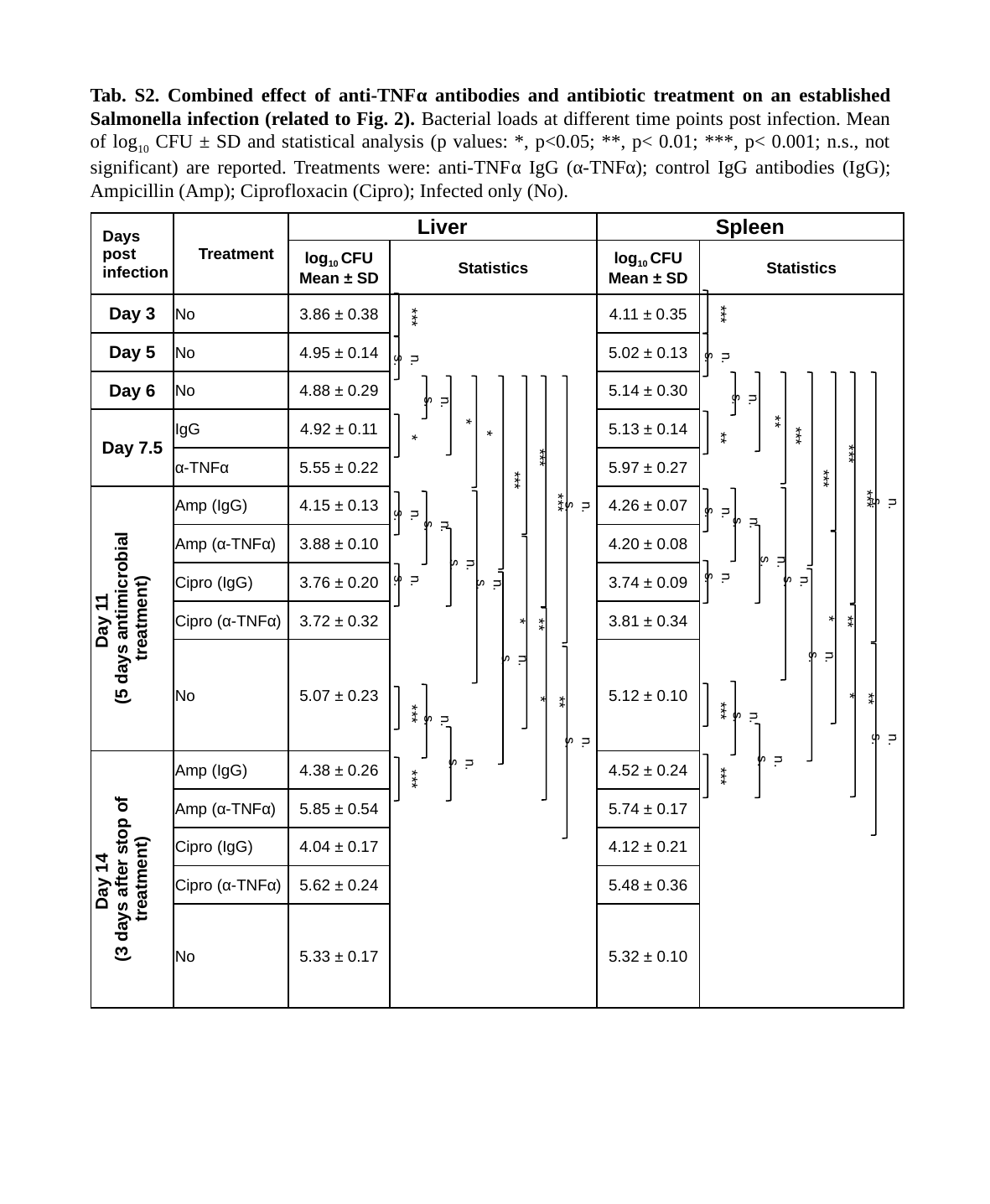

Tab. S2. Combined effect of anti-TNFα antibodies and antibiotic treatment on an established Salmonella infection (related to Fig. 2). Bacterial loads at different time points post infection. Mean of log10 CFU ± SD and statistical analysis (p values: *, p<0.05; **, p< 0.01; ***, p< 0.001; n.s., not significant) are reported. Treatments were: anti-TNFα IgG (α-TNFα); control IgG antibodies (IgG); Ampicillin (Amp); Ciprofloxacin (Cipro); Infected only (No).
| Days post infection | Treatment | Liver | | Spleen | |
| --- | --- | --- | --- | --- | --- |
| | | log10 CFU Mean ± SD | Statistics | log10 CFU Mean ± SD | Statistics |
| Day 3 | No | 3.86 ± 0.38 | | 4.11 ± 0.35 | |
| Day 5 | No | 4.95 ± 0.14 | | 5.02 ± 0.13 | |
| Day 6 | No | 4.88 ± 0.29 | | 5.14 ± 0.30 | |
| Day 7.5 | IgG | 4.92 ± 0.11 | | 5.13 ± 0.14 | |
| | α-TNFα | 5.55 ± 0.22 | | 5.97 ± 0.27 | |
| Day 11 (5 days antimicrobial treatment) | Amp (IgG) | 4.15 ± 0.13 | | 4.26 ± 0.07 | |
| | Amp (α-TNFα) | 3.88 ± 0.10 | | 4.20 ± 0.08 | |
| | Cipro (IgG) | 3.76 ± 0.20 | | 3.74 ± 0.09 | |
| | Cipro (α-TNFα) | 3.72 ± 0.32 | | 3.81 ± 0.34 | |
| | No | 5.07 ± 0.23 | | 5.12 ± 0.10 | |
| Day 14 (3 days after stop of treatment) | Amp (IgG) | 4.38 ± 0.26 | | 4.52 ± 0.24 | |
| | Amp (α-TNFα) | 5.85 ± 0.54 | | 5.74 ± 0.17 | |
| | Cipro (IgG) | 4.04 ± 0.17 | | 4.12 ± 0.21 | |
| | Cipro (α-TNFα) | 5.62 ± 0.24 | | 5.48 ± 0.36 | |
| | No | 5.33 ± 0.17 | | 5.32 ± 0.10 | |
***
***
n.s.
n.s.
n.s.
n.s.
**
*
***
*
**
*
***
***
***
***
***
***
n.s.
n.s.
n.s.
n.s.
n.s.
n.s.
n.s.
n.s.
n.s.
n.s.
n.s.
n.s.
***
***
n.s.
n.s.
***
***
***
***
n.s.
n.s.
n.s.
n.s.
n.s.
n.s.
***
***

## Slide 3
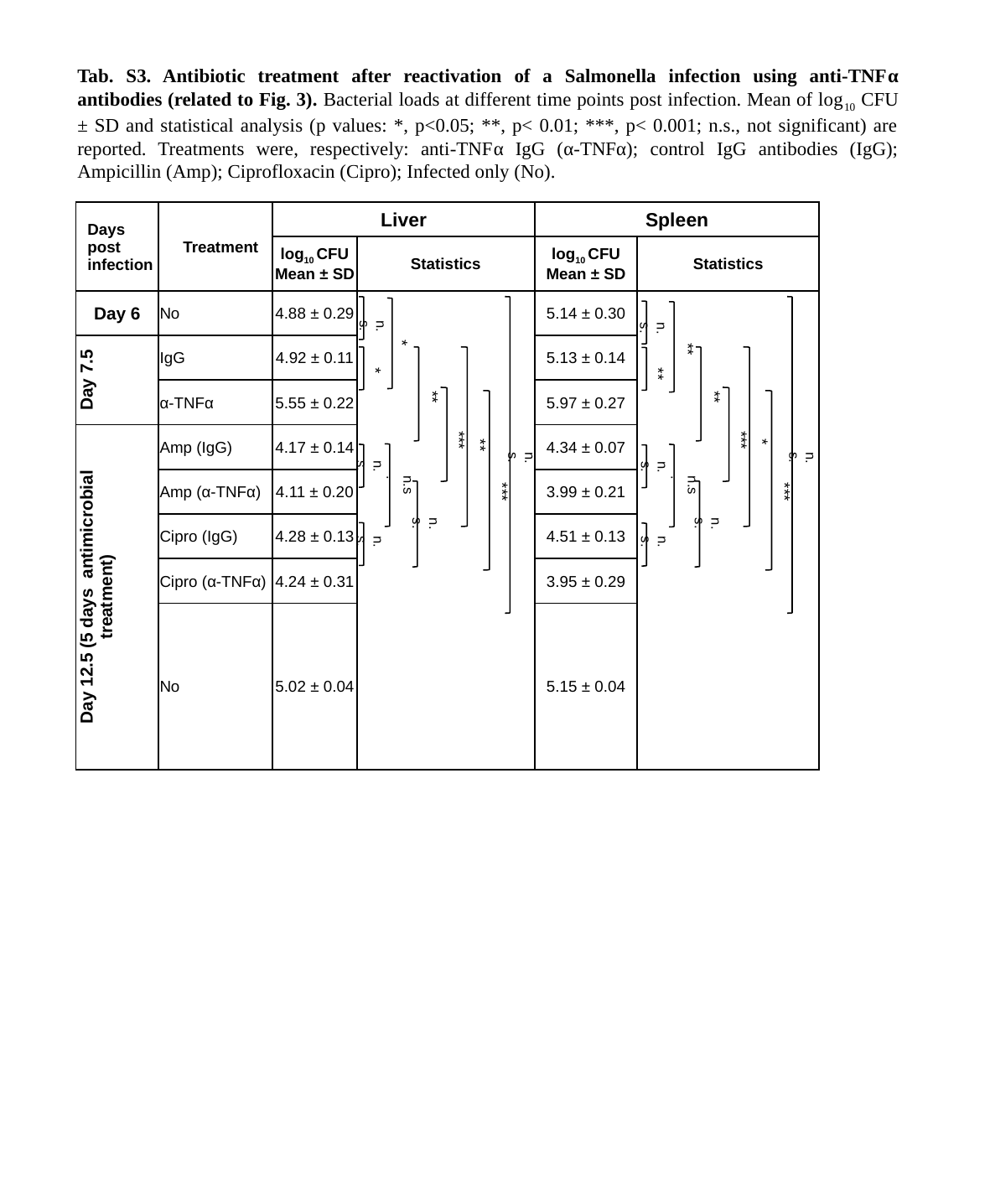

Tab. S3. Antibiotic treatment after reactivation of a Salmonella infection using anti-TNFα antibodies (related to Fig. 3). Bacterial loads at different time points post infection. Mean of log10 CFU ± SD and statistical analysis (p values: *, p<0.05; **, p< 0.01; ***, p< 0.001; n.s., not significant) are reported. Treatments were, respectively: anti-TNFα IgG (α-TNFα); control IgG antibodies (IgG); Ampicillin (Amp); Ciprofloxacin (Cipro); Infected only (No).
| Days post infection | Treatment | Liver | | Spleen | |
| --- | --- | --- | --- | --- | --- |
| | | log10 CFU Mean ± SD | Statistics | log10 CFU Mean ± SD | Statistics |
| Day 6 | No | 4.88 ± 0.29 | | 5.14 ± 0.30 | |
| Day 7.5 | IgG | 4.92 ± 0.11 | | 5.13 ± 0.14 | |
| | α-TNFα | 5.55 ± 0.22 | | 5.97 ± 0.27 | |
| Day 12.5 (5 days antimicrobial treatment) | Amp (IgG) | 4.17 ± 0.14 | | 4.34 ± 0.07 | |
| | Amp (α-TNFα) | 4.11 ± 0.20 | | 3.99 ± 0.21 | |
| | Cipro (IgG) | 4.28 ± 0.13 | | 4.51 ± 0.13 | |
| | Cipro (α-TNFα) | 4.24 ± 0.31 | | 3.95 ± 0.29 | |
| | No | 5.02 ± 0.04 | | 5.15 ± 0.04 | |
n.s.
n.s.
*
**
*
**
**
**
***
***
**
*
n.s.
n.s.
n.s.
n.s.
n.s.
n.s.
***
***
n.s.
n.s.
n.s.
n.s.
